# AGE-RAGE Axis Involvement in Allergies and Autoimmunity: Cellular Signaling, Barrier Dysfunction and Immune Polarization

**DOI:** 10.3390/biom16020241

**Published:** 2026-02-03

**Authors:** Enrica Dato, Alessandra Ventre, Marilena Di Salvo, Federica Nuccio, Marco Casciaro, Sebastiano Gangemi

**Affiliations:** 1 Department of Clinical and Experimental Medicine, Unit and School of Allergy and Clinical Immunology, University Hospital of Messina, University of Messina, 98125 Messina, Italy; enricadato98@gmail.com (E.D.); alessandraven144@gmail.com (A.V.); marilenadisalvo98@gmail.com (M.D.S.); federica.nuccio01@gmail.com (F.N.); gangemis@unime.it (S.G.); 2 Department of Medical Sciences, Unit and School of Allergy and Clinical Immunology, University Hospital of Messina, 98125 Messina, Italy

**Keywords:** advanced glycation end-products, AOPP, AGE-RAGE axis, sRAGE, oxidative stress, autoimmunity, allergy, atopy, chronic inflammation, immune dysregulation

## Abstract

Advanced glycation end-products (AGEs) are a variety of endogenous and exogenous substances that play an important role in inflammation, allergies, and autoimmune diseases. AGEs’ pathogenicity, alongside advanced oxidation protein products (AOPPs) and other ligands, lies in their ability to bind the receptor for advanced glycation end-products (RAGE) and trigger pro-inflammatory signaling pathways and cytokine release. The literature reports numerous studies on the role of the AGE-RAGE axis in various allergic conditions, including bronchial asthma, atopic dermatitis, food allergies, and autoimmune diseases such as rheumatoid arthritis, systemic lupus erythematosus, or Hashimoto’s thyroiditis, where the significant role of the AGE–RAGE axis in the immunopathogenesis of both allergic and autoimmune conditions is largely discussed and demonstrated. They suggest promising opportunities for the development of new diagnostic markers and targeted therapeutic strategies. However, further large-scale studies are needed to fully understand this multifaceted pathway and translate these insights into effective clinical interventions.

## 1. Introduction

Oxidative stress is a physiological condition that arises when there is an imbalance between the production of reactive oxygen species (ROS) and the ability of biological antioxidant defence systems to neutralize them. Under normal circumstances, ROS are continuously generated as byproducts of cellular metabolism, particularly in the mitochondrial electron transport chain. These reagents have a crucial function in cell signaling, immune response, and the maintenance of homeostasis. However, an excessive production of ROS, which may be triggered by environmental stressors, mitochondrial dysfunction, or enzymatic dysregulation, can lead to oxidative damage to lipids, proteins, and nucleic acids [1,2].

Advanced glycation end-products (AGEs) represent a heterogeneous group of both endogenous and exogenous substances that share clinical relevance in the pathogenesis of various inflammatory and autoimmune diseases such as diabetes, cardiovascular diseases, and cancer, both solid and hematological [3,4].

Endogenous AGE production represents a classic example of a three-step Maillard reaction. The first step consists of glycation, in which a carbonyl group (–C=O) of reducing sugars reacts with the amino group (–NH_2_) of proteins, nucleic acids, or lipids, resulting in the formation of a Schiff base. The second step involves the Amadori rearrangement of this early glycation product. Finally, non-enzymatic peptide crosslinking occurs, leading to the formation of stable AGEs. On the other hand, various processes could lead to the formation of exogenous AGEs, including biochemical reactions during cooking [5,6,7].

AGE production and consequent accumulation occur physiologically. However, their levels can increase under certain conditions. Oxidative stress plays a crucial role in the formation and accumulation of AGEs. The Maillard reaction can be accelerated by elevated ROS levels because they promote the oxidation of early glycation intermediates into reactive carbonyl species, such as glyoxal, methylglyoxal, and 3-deoxyglucosone. These compounds further enhance protein crosslinking and the accumulation of AGEs. On the contrary, AGEs can worsen oxidative stress by binding to their cellular receptor and activating downstream signaling pathways that generate additional ROS. This bidirectional interaction favors the creation of a self-perpetuating cycle of oxidative damage and glycoxidation [8,9,10].

AGEs’ biological significance mostly derives from the engagement of cell surface receptors, namely the receptor for advanced glycation end-products (RAGE) and its soluble forms (sRAGE) [6,11,12].

RAGE is a multiligand cell surface receptor that belongs to the immunoglobulin superfamily and is widely expressed in various tissues, including the vascular endothelium and immune cells. RAGE exists in multiple structural and functional forms, arising from alternative splicing of the AGER gene and proteolytic cleavage of the full-length receptor. The soluble receptor for advanced glycation end-products (sRAGE) acts as a decoy receptor by binding to and neutralizing pro-inflammatory RAGE ligands. This dual role has been associated with protection against inflammatory stress and related diseases. However, high levels of sRAGE may indicate sustained overstimulation of cell surface RAGE, which can worsen inflammatory processes [11,13,14].

Moreover, circulating sRAGE levels may mirror overall RAGE activity. They can rise in parallel with AGE levels as a compensatory response to counteract AGE-induced tissue damage [15,16].

In addition to AGEs, other ligands, both endogenous and exogenous, like high-mobility group box 1 (HMGB1), S100/calgranulin proteins, amyloid-β peptides and DNA bind RAGE. This interaction leads to the activation of multiple intracellular signaling pathways, notably the nuclear factor-kappa B (NF-κB), mitogen-activated protein kinase (MAPK), and Janus kinase/signal transducer and activator of transcription (JAK/STAT). The action of these pathways result in increased expression of pro-inflammatory cytokines, adhesion molecules, and mediators of oxidative stress. The sustained activation of RAGE establishes a positive feedback loop that amplifies inflammation and cellular dysfunction [5,11,17].

Among RAGE’s ligands, advanced oxidation protein products (AOPPs) are emerging as a significant marker of oxidative damage. AOPPs are substances generated when plasma proteins, especially albumin, are exposed to oxidative chlorine species, such as hypochlorous acid (HOCl), derived from the myeloperoxidase/H_2_O_2_/halide system. Once produced, AOPPs serve as mediators of inflammation and redox signaling by stimulating NADPH oxidase to produce additional ROS. They also activate NF-κB and MAPK signaling pathways and trigger the release of pro-inflammatory cytokines, such as TNF-α and IL-6. Therefore, AOPPs both reflect and amplify oxidative stress [6,18,19].

The AGE–RAGE signaling axis has emerged as a clinically relevant pathway in both allergic and autoimmune disorders, as shown in Table 1 and Table 2.

In individuals with allergies, RAGE activation contributes to dysfunction of the epithelial barrier. This facilitates allergen penetration and leads to heightened immune sensitization and Th2-mediated response. Dietary AGEs (dAGEs) can impair the integrity of the intestinal barrier and enhance type 2 immune responses by activating RAGE and toll-like receptor 4 (TLR4). This mechanism may ultimately lead to increased allergic sensitization [20,30,55].

In autoimmune diseases, the activation of the AGE-RAGE axis and the consequent activation of pathways such as NF-κB, resulting in the expression of cytokines and adhesion molecules that sustain a pro-inflammatory loop that can worsen autoimmunity and damage to end organs. These findings suggest that targeting the AGE-RAGE axis could be a potential therapeutic strategy for both allergic and autoimmune conditions [56,57,58].

The aim of this study is to conduct a comprehensive literature review of the past 10–15 years to examine the involvement of the AGE–RAGE axis in autoimmune and allergic diseases. By synthesizing current evidence, this review seeks to elucidate the underlying and potentially shared pathogenic mechanisms across these conditions. A deeper understanding of these pathways may encourage further elucidation on the use of AGE–RAGE-related factors as prognostic or disease-severity biomarkers, as well as potential therapeutic targets in future clinical applications.

## 2. Materials and Methods

We conducted a narrative literature review using PubMed (National Center for Biotechnology Information, Bethesda, MD, USA; https://pubmed.ncbi.nlm.nih.gov/) and Embase (Elsevier, Amsterdam, The Netherlands; https://www.embase.com/landing?status=yellow) to identify articles published between January 2015 and December 2025. Our search strategy included keywords such as “advanced glycation end-products,” “AOPP,” “AGE–RAGE axis,” “sRAGE,” “oxidative stress,” “autoimmunity,” “allergy,” “atopy,” “chronic inflammation,” and “immune dysregulation.” Only peer-reviewed articles published in English were considered. Studies were included if they investigated associations between the AGE–RAGE axis and allergic or autoimmune diseases. We excluded articles unrelated to immune-mediated conditions or those lacking relevant mechanistic or clinical data. All articles were screened for relevance by reviewing titles and abstracts, followed by an assessment of alignment with the review’s objectives. We evaluated study quality and potential biases by examining study design, methodology, and consistency of findings. While our primary focus was on research from the past decade, we also included select influential studies published before 2015 for their scientific significance.

## 3. AGE-RAGE Axis in Atopic Conditions

The AGE–RAGE axis plays a significant role in the pathogenesis of allergic airway diseases, particularly asthma. AGEs are generated through non-enzymatic glycation reactions between sugars and biological macromolecules, accumulating under conditions of chronic inflammation and oxidative stress. Their interaction with RAGE, which is highly expressed in pulmonary tissue, triggers pro-inflammatory signaling pathways that sustain and amplify immune responses. sRAGE acts as a natural antagonist by binding RAGE ligands and thereby limiting receptor-mediated inflammatory activation. Reduced circulating levels of sRAGE are associated with more severe inflammation, supporting its potential role as a biomarker. Overall, the AGE–RAGE axis represents a key modulator of allergic inflammation and a promising therapeutic target in allergic respiratory diseases.

### 3.1. Asthma

Recent evidence identifies RAGE activation as a pivotal driver of airway inflammation in bronchial asthma (BA) and other allergic airway diseases. Elevated levels of RAGE ligands and enhanced downstream signaling are consistently associated with more severe disease phenotypes, including neutrophilic inflammation and steroid resistance. In parallel, experimental data indicate that RAGE critically regulates type 2 (T2) immune responses, thereby contributing to the development and maintenance of T2-high BA [59].

Experimental work by Perkins et al. demonstrates that RAGE is a central modulator of IL-4- and IL-13-driven inflammatory programs within lung tissue. In RAGE-deficient models, these cytokines fail to fully induce hallmark features of T2-high BA, including airway inflammation and mucosal remodeling. These findings indicate that RAGE is required for optimal signaling through IL-4R and IL-13R, particularly within non-hematopoietic lung cells such as epithelial and stromal compartments. From a therapeutic perspective, RAGE inhibition emerges as a potential strategy to attenuate both inflammation and structural remodeling, especially in phenotypes characterized by goblet cell metaplasia [60].

The seminal study by Oczypok et al. further establishes the AGE–RAGE axis as an initiator in allergic airway inflammation [61]. Using murine models, the authors demonstrate that epithelial RAGE expression is essential for allergen-induced IL-33 production following exposure to aeroallergens such as Alternaria alternata or house dust mites. In the absence of RAGE, IL-33 induction is markedly reduced, resulting in the failure to activate downstream type 2 immune responses, including ILC2 expansion, eosinophilic inflammation, and airway hyper-responsiveness. Importantly, this mechanism is driven primarily by RAGE expressed on structural lung cells, reinforcing the role of the airway epithelium as a damage-sensing interface that translates tissue stress signals into type 2 immune activation. The association between *AGER* gene variants, particularly the *G82S* polymorphism, and increased asthma susceptibility further supports the clinical relevance of this pathway [61].

Clinical evidence supporting the regulatory role of RAGE is provided by El-Seify et al., who evaluated sRAGE levels in patients with BA. sRAGE acts as a decoy receptor that neutralizes pro-inflammatory RAGE ligands. Asthmatic patients, especially those with moderate-to-severe disease, exhibit significantly reduced serum sRAGE levels, which correlate with impaired lung function, elevated eosinophil counts, and increased total IgE levels. These findings suggest that reduced sRAGE availability facilitates sustained RAGE activation, thereby promoting chronic type 2 inflammation. Consequently, sRAGE may serve as an inverse biomarker of disease severity and inflammatory burden in asthma [21].

Finally, studies on oxidative stress, including the work by Ammar et al., highlight a pronounced redox imbalance in BA. Increased levels of oxidative damage markers, such as AOPP and malondialdehyde (MDA), are accompanied by reduced antioxidant defences, particularly in uncontrolled disease. Although these oxidative markers show limited correlation with spirometric parameters, they reflect systemic inflammatory and metabolic alterations that contribute to disease persistence. Collectively, these findings support the concept that RAGE-mediated signaling and oxidative stress converge to sustain chronic airway inflammation in BA [22].

### 3.2. Atopic Dermatitis

In recent years, increasing attention has been paid to the AGE–RAGE axis in dermatology, as growing evidence implicates this pathway in the inflammatory and oxidative mechanisms underlying atopic dermatitis (AD). In adult patients, Hong et al. demonstrated a local accumulation of AGEs within corneocytes that correlated with disease severity, while serum AGE levels remained comparable to those of healthy controls. This finding suggests a predominantly cutaneous activation of the pathway. Notably, AD patients also exhibited significantly reduced circulating levels of soluble RAGE isoforms (sRAGE and esRAGE), indicating a diminished capacity to counteract membrane-bound RAGE activation. This imbalance between local AGE accumulation and reduced systemic regulation may sustain chronic skin inflammation and contribute to the metabolic comorbidities frequently associated with AD [23].

Oxidative stress represents a closely related pathogenic mechanism capable of amplifying AGE–RAGE signaling. Excessive production of reactive oxygen species promotes lipid peroxidation, generating bioactive aldehydes that disrupt cellular membranes and impair skin barrier integrity, thereby perpetuating inflammation. Secondary lipid peroxidation products, including malondialdehyde and 4-hydroxynonenal, not only serve as markers of oxidative damage but also actively enhance inflammatory signaling, establishing self-perpetuating loops between oxidative stress and immune activation. These processes are particularly relevant in inflammatory dermatoses, where barrier dysfunction and immune dysregulation are tightly interconnected [24].

Among oxidative stress markers, MDA is one of the most extensively studied in allergic diseases. Elevated MDA levels have been consistently reported in asthma, atopic dermatitis, allergic rhinitis, and food allergy, often correlating with disease severity. Beyond its role as a biomarker, MDA exerts biological effects through the formation of adducts with proteins and membrane lipids, contributing to epithelial barrier dysfunction, immune activation, and chronic inflammation. Accordingly, MDA represents a key molecular link between lipid peroxidation, oxidative stress, and allergic inflammation [25].

Evidence from pediatric populations further supports the relevance of AGE–RAGE dysregulation in AD. Eke-Gungor et al. reported significantly reduced serum sRAGE levels in children with AD compared with healthy controls, independent of disease severity, total IgE levels, or eosinophilia. This reduction, interpreted as a loss of a neutralizing mechanism for pro-inflammatory RAGE ligands such as AGEs, HMGB1, and S100 proteins, may promote enhanced RAGE-mediated signaling and a pro-oxidative, pro-inflammatory environment from early stages of disease. Although sRAGE does not currently appear to be a severity biomarker, it may represent an indicator of susceptibility and a regulator of inflammatory homeostasis in atopic skin [26,62].

A broader perspective is provided by Guarneri et al., who reviewed the involvement of the AGE–RAGE system across major inflammatory and infectious skin diseases. The authors describe how AGE binding to RAGE on keratinocytes, fibroblasts, and endothelial cells activates NF-κB-dependent pathways, leading to cytokine release and increased oxidative stress, thereby reinforcing chronic inflammation and tissue damage. Reduced sRAGE levels are frequently associated with greater disease activity, supporting the concept of RAGE modulation as a potential therapeutic strategy. However, the relationship between AGE–RAGE dynamics and chronological aging remains insufficiently defined [17].

Overall, available evidence indicates that in both adult and pediatric AD, the AGE–RAGE axis is skewed toward pro-inflammatory activation, characterized by tissue AGE accumulation and reduced soluble RAGE forms. This imbalance compromises control of inflammatory and oxidative signaling, contributing to disease persistence and possibly to metabolic comorbidities. Modulation of the AGE–RAGE pathway, either by restoring sRAGE levels or inhibiting membrane-bound RAGE, therefore emerges as a promising therapeutic and translational research avenue.

Finally, oxidative protein damage represents an additional component of redox imbalance in AD. AOPPs are consistently elevated in AD patients, reflecting increased protein-targeted oxidative stress. AOPP accumulation has been proposed to contribute to skin barrier dysfunction and pro-inflammatory signaling; however, current evidence remains limited and methodologically heterogeneous. Consequently, it is still unclear whether AOPPs play an active pathogenic role or primarily serve as biomarkers of ongoing inflammation and oxidative stress, highlighting the need for further well-designed studies [27].

### 3.3. Food Allergy

Over recent decades, the rising prevalence of food allergies has paralleled the widespread adoption of diets rich in sugars, ultra-processed foods, and high-temperature cooking products. In this context, Smith et al. proposed that dAGEs may contribute to allergic disease development through activation of the AGE–RAGE axis [28]. AGEs can accumulate endogenously or be introduced exogenously via fried, grilled, and heavily processed foods. Upon binding to RAGE, which is expressed on epithelial, endothelial, and immune cells, AGEs activate pro-inflammatory signaling pathways, including NF-κB, leading to cytokine release and increased oxidative stress.

According to this model, dAGEs compromise intestinal barrier integrity by disrupting epithelial tight junctions and increasing mucosal permeability, thereby facilitating antigen translocation and allergic sensitization. Beyond barrier dysfunction, AGE–RAGE interactions modulate immune responses by promoting Th2 polarization and the production of IL-4, IL-5, and IL-13. Concomitant activation of RAGE and innate immune receptors such as TLR4 further amplifies allergic inflammation. In this context, sRAGE may exert a protective role by acting as a decoy receptor, with higher circulating levels associated with reduced IgE responses. Additionally, AGE-rich diets adversely affect the gut microbiota, reducing butyrate-producing bacteria essential for epithelial integrity and immune tolerance, thereby reinforcing inflammation and susceptibility to sensitization [28].

Mechanistic support for this hypothesis is provided by Zhang et al., who demonstrated a direct link between dAGE exposure and food allergy development through coordinated activation of the RAGE–TLR4 axis [20].

Their epidemiological analysis showed that diets rich in ultra-processed foods, and consequently high in AGEs, are associated with increased prevalence of allergic disorders, particularly food allergy. In experimental models, high-dAGE diets increased intestinal permeability, enhanced Th2 immune responses, and elevated allergen-specific IgE levels. Genetic ablation of either RAGE or TLR4 almost completely abolished the adjuvant effect of dAGEs, confirming the central role of this molecular pathway. Consistent findings in human immune cells further supported the translational relevance of these observations [20].

Complementary evidence is provided by Del Giudice et al., who reviewed epidemiological and pathophysiological data linking high consumption of ultra-processed foods to increased risk of allergic and respiratory diseases in childhood, identifying activation of the AGE–RAGE axis as a key pathogenic mechanism [29]. Chronic stimulation of this pathway promotes oxidative stress, epithelial barrier dysfunction, and immune deviation toward a Th2 phenotype. Oxidative damage, partly mediated by lipid peroxidation products such as malondialdehyde and 4-hydroxynonenal, further amplifies inflammatory signaling and barrier impairment [63].

Together with diet-induced dysbiosis, these mechanisms contribute to sustained low-grade inflammation and loss of mucosal immune tolerance. Although current evidence remains largely observational, the authors suggest that reducing dietary exposure to AGEs may represent a plausible preventive strategy to preserve mucosal integrity and limit allergic disease susceptibility during childhood [29].

## 4. AGE–RAGE Axis in Autoimmune Diseases

The AGE–RAGE axis plays a key role in several autoimmune diseases by promoting chronic inflammation, oxidative stress, and tissue damage. In Systemic Lupus Erythematosus (SLE), it enhances pro-inflammatory cytokine production and contributes to renal injury. In rheumatoid arthritis (RA), it drives synovial proliferation, inflammatory cell infiltration, and joint destruction. In type 1 diabetes, AGE–RAGE signaling exacerbates autoimmune β-cell damage, and in Hashimoto’s thyroiditis (HT), it may contribute to thyroid tissue injury and the autoimmune response. In multiple sclerosis (MS), the axis amplifies neuroinflammation and disrupts the blood–brain barrier. In psoriasis, AGE accumulation and RAGE activation enhance keratinocyte activity and the Th17 cytokine network; in Sjögren’s Syndrome, activation of the AGE/RAGE pathway and potentially reduced sRAGE contribute to immune dysfunction and glandular damage. In Systemic Sclerosis, sRAGE alterations play a specific role in pulmonary complications, exhibiting opposite profiles in pulmonary arterial hypertension (PAH) versus interstitial lung disease (ILD). Together, these findings indicate a shared mechanistic framework with disease-specific patterns across distinct autoimmune conditions. AGE–RAGE forms a self-perpetuating inflammatory loop that worsens tissue injury in autoimmune conditions. We aimed to examine the role of the AGE–RAGE axis in the pathogenesis of these autoimmune diseases.

### 4.1. AGE–RAGE Axis in Rheumatoid Arthritis

The AGE–RAGE axis is a central mechanism in chronic inflammation and autoimmune/rheumatic diseases. AGE and other oxidative ligands can bind to RAGE on endothelial, synovial, and immune cells, activating pro-inflammatory pathways, while sRAGE can sequester these ligands, acting as a decoy and limiting receptor activation [31].

In RA patients, Nadali et al. [32] show that low plasma sRAGE levels precede and predict cardio-metabolic events, suggesting that reduced decoy receptor capacity may contribute to comorbidities [32]. In contrast, Nakhjavani et al. report elevated sRAGE in RA patients compared with healthy controls, which positively correlates with disease activity, highlighting variable behavior across clinical contexts [33].

Mechanistically, Lou et al. demonstrated that exposure of synovial fibroblasts to AOPPs activates the RAGE—NF-κB pathway, promoting inflammatory responses and invasive cellular behavior, confirming the pathogenic role of RAGE in joints [30]. Park et al. showed in a murine model that overexpression of sRAGE in mesenchymal stromal cells reduces joint inflammation, decreases Th17 cells, and increases T-reg cells, indicating a protective and immunomodulatory potential of sRAGE [34].

The review by Delrue et al. [31] emphasizes that the variability in sRAGE levels across studies reflects methodological differences, disease stage, the type of ligands measured, and comorbidities, making it difficult to draw definitive conclusions regarding the protective or harmful role of the AGE–RAGE/sRAGE axis [31].

Overall, all five studies confirm that the balance between AGE/AOPP ligands and sRAGE is crucial for modulating inflammation and cardio-metabolic complications. Clinical and experimental results are heterogeneous and sometimes conflicting, highlighting the need for longitudinal, standardized studies with detailed sRAGE and AGE measurements to clarify their prognostic and therapeutic role in autoimmune diseases.

### 4.2. AGE-RAGE Axis in Systemic Lupus Erythematosus

The AGE–RAGE axis plays a significant role in the pathogenesis of SLE, modulating inflammation, tissue damage, and disease activity. The studies considered show that SLE patients exhibit AGE accumulation and sRAGE dysregulation, although results vary across models and methodologies. Nowak et al. report significantly increased serum levels of AGEs (CML, CEL, pentosidine) and decreased sRAGE in SLE patients, suggesting a potentially pro-inflammatory imbalance [35]. Similarly, the study on serum and skin sRAGE by Carrión-Barberà et al. confirms elevated AGE levels and shows that the AGE/sRAGE ratio correlates with disease activity and damage, highlighting the importance of the ligand–soluble isoform balance [36].

From a mechanistic perspective, the review by Delrue et al. emphasizes that RAGE, binding AGEs and other pro-inflammatory ligands (S100, HMGB1), activates NF-κB and MAPK pathways, promoting cytokine production and oxidative stress, providing a framework consistent with patient observations [31]. The experimental study by Yue et al. demonstrates, at the cellular level, that sRAGE modulation reduces inflammation and tissue damage, suggesting a protective role for the soluble isoform [37]. Finally, Watanabe et al., using lupus-prone RAGE knock-out mice, showed that RAGE deficiency attenuates lupus nephritis, with reduced proteinuria, improved histological scores, and lower neutrophil infiltration, confirming the receptor’s pathogenic role [38].

All studies confirm that the AGE–RAGE/sRAGE axis is central in SLE. Still, contradictory findings emerge, while AGE accumulation is consistently associated with inflammation and damage, the role of sRAGE varies across contexts, experimental models, and methodologies. The standard message is that not only absolute values but especially the AGE/sRAGE ratio represent relevant disease indicators and potential therapeutic targets if studied longitudinally and in a standardized manner.

### 4.3. AGE-RAGE Axis in Hashimoto’s Thyroiditis

In Hashimoto’s thyroiditis, recent studies have evaluated AGE and sRAGE in relation to thyroid status and therapy. Ruggeri et al. observed that euthyroid patients not on replacement therapy had significantly higher serum AGE and significantly lower sRAGE compared with healthy controls; the AGE/sRAGE ratio was roughly three times higher in patients, indicating a pro-oxidative/pro-inflammatory imbalance [39].

Csiha et al. reported different results in patients receiving levothyroxine therapy: mean AGE levels were slightly lower and sRAGE higher than in controls, with a lower median AGE/sRAGE ratio. In this study, sRAGE negatively correlated with TSH, and AGE negatively correlated with fT3, suggesting that adequate replacement therapy and reasonable metabolic control can modulate the balance between AGE and sRAGE [40].

The study by Punda et al. measured tissue AGE and catestatin levels in patients with Hashimoto’s disease and healthy controls. AGE levels were significantly higher in patients and were positively correlated with catestatin and weakly correlated with inflammatory markers such as hs-CRP and anti-TPO antibodies. This study did not include sRAGE, but it provides additional evidence of increased advanced glycation products in patients [41].

In conclusion, these studies indicate that AGE levels can be elevated in Hashimoto’s thyroiditis, whereas sRAGE levels vary with thyroid status and replacement therapy. The increase in tissue AGE suggests a potential role in oxidative stress and inflammation, whereas including sRAGE as a serum marker may provide further information on patients’ metabolic and thyroid conditions.

### 4.4. AGE–RAGE Axis in Type 1 Diabetes

Available evidence supports a multifaceted involvement of the AGE–RAGE axis in type 1 diabetes (T1D), encompassing immune modulation, tissue microenvironment alterations, and the development of diabetes-related damage. Across experimental and human studies, increased AGE accumulation and enhanced RAGE expression are consistently associated with pro-inflammatory signaling and tissue injury [42,43]. In immunological models, RAGE has been shown to influence T-cell survival and activation, supporting its role in sustaining inflammatory responses relevant to T1D pathogenesis [44,45,46].

However, the behavior of soluble RAGE isoforms appears more variable and context-dependent. Observational studies report divergent modulation of AGEs and sRAGE in response to metabolic conditions such as fasting and feeding [47], higher circulating sRAGE levels have also been associated with lipid-lowering therapy, indicating that pharmacological and metabolic factors can significantly influence circulating biomarkers independently of disease activity [48]. Experimental interventions further suggest that modulation of the AGE/RAGE axis may be achievable, as antioxidant supplementation combined with insulin reduces AGE accumulation and downregulates tissue RAGE expression, ameliorating structural and functional damage [42]. Nevertheless, these findings are derived from animal models and their translational relevance to human T1D remains uncertain. In addition, several studies focus on downstream complications and interconnected signaling pathways, including cardiac and renal fibrosis as well as Wnt/β-catenin and Klotho signaling, adding further complexity to the interpretation of AGE/RAGE involvement [43].

These data indicate that while the AGE/RAGE axis represents a relevant mechanistic contributor to immune dysregulation and tissue injury in T1D, its biomarker readouts—particularly sRAGE and its isoforms—are highly sensitive to experimental design, metabolic state, and therapeutic exposure. This heterogeneity limits the interpretability of circulating AGE–sRAGE measures in isolation and underscores the need for standardized clinical studies that clearly define molecular targets, measurement strategies, and modulating interventions [42,43,44,45,46,47,48].

### 4.5. AGE–RAGE Axis in Psoriasis

The three studies collectively highlight the pivotal role of the AGE/RAGE axis and its soluble form, sRAGE in psoriasis, supporting their relevance as both biomarkers and mechanistic contributors to disease pathogenesis. In the case–control analysis by Karas et al., psoriatic patients exhibited significantly elevated serum levels of AGEs and RAGE, together with reduced NAD concentrations [49]. These findings were interpreted as evidence of sustained systemic inflammation and accelerated inflammaging, consistent with enhanced oxidative stress and ROS production typical of psoriasis. Similarly, Damasiewicz-Bodzek and Nowak found markedly higher levels of CML, CEL, and sRAGE in both active and remission phases. Although these markers decreased upon remission, they remained significantly above control values, indicating persistent glycoxidation processes and ongoing RAGE-pathway activation even after clinical improvement [50].

The mechanistic study by Kang et al. provides essential experimental insight, demonstrating that AGEs accumulate in psoriatic keratinocytes and activate RAGE, triggering enhanced proliferation, K17 induction, and IL-36α production through STAT1/3 signaling [51]. AGE-stimulated IL-36α further promotes Th17 responses, thereby reinforcing the psoriatic cytokine circuit. These findings connect elevated AGEs not only to systemic inflammatory activity but also to the direct priming of keratinocytes—key drivers of cutaneous innate immunity in psoriasis.

The three studies converge in showing that AGEs, RAGE, and sRAGE are consistently altered in psoriasis and reflect both metabolic and immune dysregulation. Their combined clinical and mechanistic significance supports their potential role as reliable biomarkers and as meaningful factors in the interplay between chronic inflammation, metabolic disturbance, and psoriatic pathogenesis.

### 4.6. AGE–RAGE Axis in Multiple Sclerosis

Available evidence indicates a heterogeneous involvement of the AGE/RAGE axis in multiple sclerosis (MS), with findings that are partly consistent but also divergent from those reported in other chronic inflammatory conditions such as psoriasis. In the study by Rahimi et al., patients with relapsing–remitting MS receiving stable IFNβ-1a therapy showed significantly increased RAGE transcript levels and higher plasma sRAGE levels that healthy controls [52]. Notably, sRAGE showed an inverse correlation with disability (EDSS), suggesting a potentially protective or compensatory function. In contrast, Damasiewicz-Bodzek et al. found only slightly elevated AGE levels and slightly reduced sRAGE concentrations in MS patients, with no statistically significant differences and no associations with disease duration or disability [53]. These discordant findings indicate that alterations of the AGE/RAGE axis in MS are subtle and highly dependent on clinical context, including disease stability and therapeutic exposure. Rather than reflecting a consistent disease-driven dysregulation, sRAGE levels in MS may capture dynamic compensatory mechanisms or treatment-related effects, which limits their reliability as a standalone biomarker of disease activity or barrier dysfunction.

### 4.7. AGE-RAGE in Systemic Sclerosis and Sjögren’s Syndrome

The two studies analyzed provide important and partly distinct insights into the involvement of the AGE/RAGE axis and its soluble form, sRAGE, in Systemic Sclerosis (SSc) and Sjögren’s Syndrome (pSS). Rather than indicating a uniform pathogenic role, the available evidence suggests that the AGE/RAGE axis may exert disease-specific effects across autoimmune connective tissue disorders. The review by Delrue et al. describes how activation of the AGE–RAGE pathway contributes to inflammatory and vascular processes across several rheumatic diseases, including pSS [31]. The authors highlight that RAGE is expressed in innate immune and endothelial cells, and that its activation promotes pro-inflammatory cytokine release, while low sRAGE levels—as reported in multiple autoimmune contexts—reduce the capacity to neutralize AGEs, thereby perpetuating chronic inflammation. In pSS, although disease-specific quantitative data are lacking, the reported association between AGE/RAGE signaling and immune dysfunction suggests a predominantly pro-inflammatory role of this pathway. In this context, reduced sRAGE availability may further amplify oxidative stress and contribute to glandular tissue injury by limiting the decoy function against AGEs.

In Systemic Sclerosis, the role of the AGE/RAGE axis is defined more precisely in the prospective study by Atzeni et al. [54].

The authors demonstrate that serum sRAGE levels vary markedly depending on pulmonary phenotype: they are significantly increased in patients with pulmonary arterial hypertension (SSc-PAH) and significantly decreased in those with interstitial lung disease (SSc-ILD). Moreover, high baseline sRAGE concentrations predicted future development of PAH and PAH-related mortality, suggesting both pathogenetic and prognostic relevance. This phenotype-dependent divergence indicates a more complex and context-specific role of sRAGE in SSc, potentially reflecting vascular remodeling or endothelial stress rather than a simple deficiency of its decoy activity. This bidirectional behavior of sRAGE in SSc contrasts with the generally reduced levels reported in other autoimmune diseases and underscores the need for a disease- and phenotype-specific interpretation of this biomarker.

Taken together, these findings highlight that while the AGE/RAGE axis is implicated in both SSc and pSS, the underlying pathogenic mechanisms differ substantially. pSS appears to be mainly characterized by immune-driven inflammatory activation and potential sRAGE deficiency, whereas in SSc, sRAGE levels diverge across distinct pulmonary phenotypes, reflecting the predominance of vasculopathy and fibrotic lung involvement.

## 5. Conclusions

**Figure 1:**
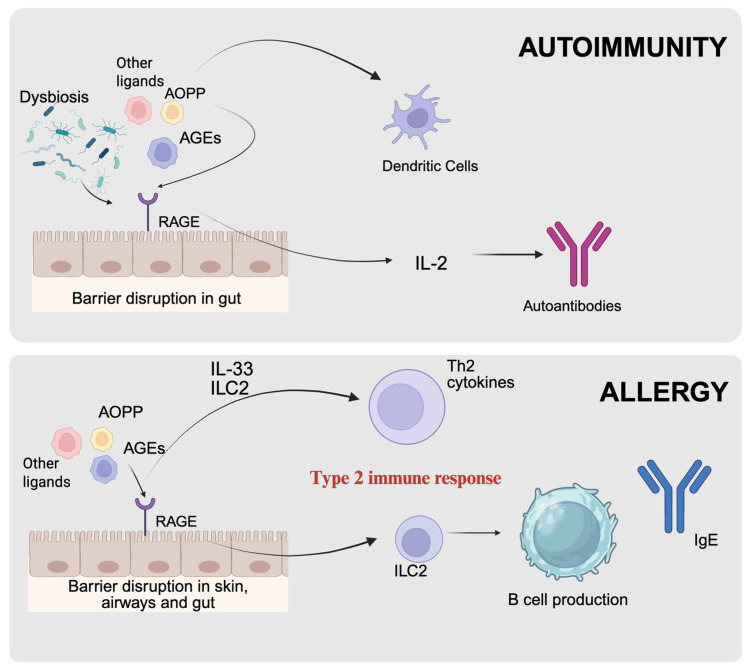
Role of the AGE–RAGE axis in autoimmunity and allergy. The accumulation of AGEs, AOPP and other RAGE ligands, promoted by dysbiosis and oxidative stress, contributes to epithelial barrier dysfunction in the gut, skin and airways. In autoimmunity, RAGE activation at the intestinal level supports dendritic cell activation, pro-inflammatory cytokine production and the development of autoantibody responses. In allergic conditions, the same signaling axis favors a type 2 immune response, driven by IL-33 and ILC2 activation, leading to Th2 cytokine production and IgE synthesis. Overall, the AGE–RAGE axis emerges as a shared mechanistic link between metabolic imbalance, barrier impairment and immune dysregulation. Created in BioRender. Nuccio, F. (2026) https://BioRender.com/okss0iv (accessed on 19 January 2026).

The findings suggest that the AGE-RAGE axis serves as a crucial inflammatory hub in both allergic and autoimmune disorders (Figure 1). Its role is not uniform; instead, it varies significantly depending on the specific immunological environment and the tissues involved.

In allergic diseases, the AGE-RAGE axis primarily facilitates the onset and amplification of type 2-driven immune responses, characterized by the activation of Th2 cells and the production of associated cytokines. This process participates in the development of allergic inflammation and often leads to barrier dysfunction, which manifests in conditions such as asthma, allergic rhinitis, and eczema.

In autoimmune disorders, the AGE-RAGE axis is more closely associated with the persistence of chronic inflammation and ongoing organ damage. This relationship highlights the axis’s role in autoimmune mechanisms that can lead to tissue injury and organ dysfunction. In summary, clarifying the diverse contributions of the RAGE–AGE axis is essential to deepen our understanding of immune dysregulation and advance innovative interventions targeting both allergies and autoimmune disorders.

## 6. Overview of Main Results

- AGEs are a group of substances that, upon binding to their receptor RAGE, activate downstream signaling pathways and aggravate disease progression of various inflammatory and autoimmune diseases.
- sRAGE acts as a decoy receptor by binding to and neutralizing pro-inflammatory RAGE ligands.
- In allergies, RAGE enhances Th2 responses and related pathways.
- In autoimmune diseases, the AGE-RAGE axis sustains chronic inflammation and organ damage, while sRAGE works as a protective factor.

## Figures and Tables

**Table 1:** Summary of methods, materials (and their sources) and conditions examined in the studies included in the review regarding allergies.

Reference	Methods	Material	Source	Condition	Object
[17]	IHC, ELISA, IF	serum	human	AD	AOPP, AGE-RAGE
[20]	ELISA, PCR, FLOW CYTOMETRY	serum	human, mice	FA	AGE-RAGE
[21]	ELISA	serum	human	BA	sRAGE
[22]	ELISA	serum	human	BA	AOPP, AGE-RAGE
[23]	IHC, ELISA	serum, skin	human	AD	AGE-RAGE
[24]	IHC, ELISA, IF	serum, skin	human, mice	AD	AOPP, AGE-RAGE
[25]	HPLC, TBA	serum	human	AD	AGE-RAGE
[26]	ELISA	serum	human	AD	sRAGE
[27]	IHC, ELISA, IF	serum	human	AD	AOPP, AGE-RAGE
[28]	IHC, ELISA, IF	serum	human	FA	AGE-RAGE
[29]	IHC, ELISA, PCR	serum	human	FA	AGE-RAGE

IHC = immunohistochemistry, ELISA = enzyme-linked immuno sorbent assay, PCR = polymerase chain reaction, IF = immunofluorescence, HPLC = high-performance liquid chromatography, BA = bronchial asthma, AD = atopic dermatitis, FA = food allergy.

**Table 2:** Summary of methods, materials (and their sources) and conditions examined in the studies included in this review regarding autoimmune diseases. IHC = Immunohistochemistry, ELISA = enzyme-linked immuno sorbent assay, PCR = polymerase chain reaction, IF = immunofluorescence, WB = Western blot; RA = rheumatoid arthritis, SLE = systemic lupus erythematosus, HT = Hashimoto’s thyroiditis; DM1 = diabetes mellitus type 1, PD = psoriasis disease, MS = multiple sclerosis, SS = Systemic Sclerosis.

Reference	Methods	Material	Source	Condition	Object
[30]	WB, IF	serum	human	RA	AOPP
[31]	IHC	synovial tissues, serum	human	RA, Sjogren	AGE-RAGE, sRAGE
[32]	ELISA	serum	human	RA	sRAGE
[33]	ELISA	serum	human	RA	sRAGE
[34]	PCR, IHC	joint tissues	mice	RA	sRAGE
[35]	ELISA	serum	human	SLE	AGE-RAGE, sRAGE
[31]	IHC	serum	human	SLE	AGE-RAGE, sRAGE
[36]	ELISA	serum	human	SLE	AGE-RAGE, sRAGE
[37]	IHC	serum	human	SLE	AGE-RAGE
[38]	PCR, IF	blood cells	mice	SLE	AGE-RAGE
[39]	ELISA	serum	human	HT	AGE-RAGE, sRAGE
[40]	ELISA	serum	human	HT	AGE-RAGE, sRAGE
[41]	ELISA	serum	human	HT	AGE-RAGE
[42]	ELISA, IHC	saliva, submandibular gland	rats	DM1	AGE-RAGE
[43]	ELISA, PCR	serum, urine	rats	DM1	AGE-RAGE
[44]	FLOW CYTOMETRY	serum	human	DM1	AGE-RAGE
[45]	ELISA, PCR	serum	human	DM1	AGE-RAGE
[46]	IF	pancreatic islets	human	DM1	AGE-RAGE
[47]	ELISA	serum	human	DM1	AGE-RAGE
[48]	ELISA	serum	human	DM1	sRAGE, AGE-RAGE
[49]	ELISA	serum	human	PD	AGE-RAGE
[50]	ELISA	serum	human	PD	sRAGE
[51]	ELISA	serum	human	PD	AGE-RAGE
[52]	ELISA, PCR	serum	human	MS	AGE-RAGE, sRAGE
[53]	ELISA	serum	human	MS	AGE-RAGE, sRAGE
[54]	ELISA	serum	human	SS	sRAGE

## Data Availability

No new data were created or analyzed in this study.

## Abbreviations

The following abbreviations are used in this manuscript:

AGE(s)	Advanced glycation end-product(s)
AOPP(s)	Advanced oxidation protein product(s)
RAGE (s)	Receptor of advanced glycation end-product(s)
ROS	Reactive oxygen species
sRAGE	Soluble receptor for advanced glycation end-product(s)
HMGB1	High-mobility group box 1
NF-kB	Nuclear factor-kappa B
MAPK	Mitogen-activated protein kinase
JAK/STAT	Janus kinase/signal transducer and activator of transcription
HOCl	Hypochlorous acid
dAGEs	Dietary AGEs
TLR4	Toll-like receptor 4
IL-4R	IL-4 receptors
IL-13R	IL-13 receptor
AD	Atopic dermatitis
MDA	Malondialdehyde
UPFs	Ultra-processed foods
SLE	Systemic Lupus Erythematosus
RA	Rheumatoid arthritis
HT	Hashimoto’s thyroiditis
MS	Multiple sclerosis
PAH	Pulmonary arterial hypertension
ILD	Interstitial lung disease
SSc	Systemic Sclerosis
pSS	Sjögren’s Syndrome
SSc-PAH	Pulmonary arterial hypertension
SSc-ILD	Interstitial lung disease
